# Sensitization to Lanolin in North-Eastern Italy, 1997–2021: Prevalence, Risk Factors and the Impact of Occupation

**DOI:** 10.3390/life14080916

**Published:** 2024-07-23

**Authors:** Luca Cegolon, Francesca Larese Filon, on behalf of the North-East Research Group on Contact Dermatitis

**Affiliations:** 1 Department of Medical, Surgical & Health Sciences, University of Trieste, 34128 Trieste, Italy; larese@units.it; 2 Public Health Department, University Health Agency Giuliano-Isontina (ASUGI), 34148 Trieste, Italy; 3 Occupational Medicine Unit, University Health Agency Giuliano-Isontina (ASUGI), 34148 Trieste, Italy

**Keywords:** contact dermatitis, lanolin alcohol, Amerchol L-101, patch test, stasis dermatitis, leg ulcer, occupational risk

## Abstract

**Background**: Direct skin contact with items containing lanolin can induce sensitization and development of contact dermatitis (CD). This multi-centric study investigated prevalence of lanolin sensitization among 30,269 outpatients from North-Eastern Italy patch tested during 1997–2021. **Methods**: European baseline and extended Triveneto series were applied on the upper part of patients’ back and removed after 48 h. Risk factors for lanolin sensitization were investigated by multiple logistic regression analysis, reporting adjusted odds ratios (aOR) with 95% confidence interval (95%CI). **Results**. Overall lanolin patch test positive ratio (PTPR) was 1.64% (=501/30,629), with variability over time and by research center. The body area most frequently affected by CD were hands (36.32%), followed by face (19.52%) and legs (8.09%), with a lanolin PTPR of 1.68%, 1.37% and 3.07%, respectively. Prevalence of occupational CD was 8.24%, and 1.83% patients with occupational CD patch tested positive against lanolin. Lanolin sensitization was significantly higher in males (aOR = 1.34; 95%CI: 1.08; 1.65) and among patients with leg CD aged 49–60 years (aOR = 2.34; 95%CI: 1.20; 4.57) or older than 60 (aOR = 4.21; 95%CI: 2.59; 6.85). Sub-group analysis confirmed the significantly higher sensitization rate of older patients with leg CD, with much stronger effect size in females 61+ years old (aOR = 5.33; 95%CI 2.87; 9.89) than males in the same age group (aOR = 2.92; 95%CI: 1.34; 6.39). Moreover, female house painters were more likely to test positive to lanolin. **Conclusions**: The variability of lanolin PTPR over time and by research center endorsed the ongoing debate on the relevance of the respective skin reaction. Clinicians assessing patients with dermatitis should collect information on potential risk factors for lanolin sensitization, particularly use of skin care products containing the hapten. Occupational exposure to lanolin-containing varnishes should also be considered.

## 1. Introduction

Lanolin is a complex hydrophobic blend of high-molecular-weight esters, accounting for 87% crude lanolin [1,2], 11% free elements (aliphatic alcohols, sterols, fatty acids and hydrocarbons) and 2% unidentified components [3]. Given the predominance of high-molecular-weight esters, lanoline is classified as a wax rather than a fat [4,5,6].

Crude lanolin (wool wax) is secreted by the sebaceous glands of sheep and can be extracted from the respective wool with a complex chemo-physical procedure [6]. In 1882, Otto Braun first patented a method to obtain pure lanolin by centrifuging the scouring liquid from wool washings. As a result, the term ‘‘lanolin’’ was coined by combining the Latin words “*lana*” (wool) and “*oleum*” (oil) [4].

Different derivatives used as personal care products can be obtained from crude lanolin: lanolin oil, lanolin wax, lanolin acid, lanolin alcohol, acetylated lanolin, acetylated lanolin alcohol, hydrogenated lanolin; hydroxylated lanolin, among others [4,7].

The emollient and hydrophobic features of lanolin and its derivatives—effective at repelling water from sheep—are used in skin care products to soften/moisturize the epidermidis, emulsify water/oil, stimulate skin gas exchange, facilitate wound healing, prevent infections and lead cosmetics and pharmaceuticals inside the skin [6,7,8,9]. The property of lanolin to penetrate deep into the epidermidis is used in topical formulations (creams, shampoos, among others) to treat various skin conditions (eczema, xerosis, inflammation, atopic/dry skin, nipple care) [10,11,12,13]. Lanolin derivatives are also used as polish furniture, or to provide water resistance and softness to leather clothes/shoes or prevent corrosion on metal surfaces or ink crystallization [14]. Lanolin has also been found in paper within leather-bound books [15].

Lanolin was first indicted as a potential allergen in 1922 in a patient with a ‘‘skin reaction’’ to a cream containing wool alcohols [16]. Several case reports of potential lanolin allergy to an ointment containing 6% wool were reported thereafter [16], until the first positive patch test was performed in 1929 [17].

There have been several attempts to identify the potential allergens of lanolin [3,18,19] and now a consensus has been reached on the free alcoholic fraction—particularly alkane-α,β-diols and alkane-α,ω-diols—produced by hydrolysis of lanolin [14], albeit other haptens may be generated by its oxidation [20]. There is evidence that hydrogenated lanolin is more allergenic than lanolin alcohol—the hapten included in the European patch test standard series [4]—whereas lanolin wax, lanolin acid and lanolin esters seem to be featured by lower patch test reactivity [4].

For instance, among 487 (6.0% = 487/8093) patients with contact dermatitis (CD) due to cosmetics, patch tested during May 1977–September 1980 by the North American Contact Dermatitis Group (NACDG), 7 turned out positive against crude lanolin, 11 to lanolin alcohol, and 2 to lanolin oil, for a final number of 20 (4.1%) reactions against lanolin or its respective components [21].

Nonetheless, the relevance of lanolin as a hapten and the significance of the respective skin reaction has been debated for decades, due to the risk of false positive results attributable to different lanolin derivatives employed in patch test formulations [4,9,13,14,22,23]. The lanolin patch test positive ratio (PTPR) in fact varied between 0.45–6.29% according to different epidemiological studies conducted at different time periods, and this variability seems to be explained by different patch test formulations [4,9,10,24,25,26,27]. This lack of consensus on relevance of lanolin PTPR increased more recently, following studies contrasting lanolin alcohol 30% against Amerchol L-101 50%, a formulation reportedly more reactive than the former, increasing the suspicion of possible false positive reactions [4,9,14,22,26,28,29,30]. As mentioned above, lanolin alcohol 30% pet. is the standard patch test formulation included in the European standard series since 1969 [28]. By contrast, Amerchol L-101 50% is a mixture of 10% lanolin alcohols and mineral oil [30,31].

However, in view of a higher PTPR, Amerchol L-101 50% was included in 2011 in NACDG screening series [10,26]. In a North-American study conducted on 43,691 patients during 2001–2018, lanolin PTPR was 3.27%, increasing from 2.16% during years 2001–2011 (when lanolin alcohol 30% pet. was used) to 4.63% in 2011–2018 (when Amerchol^®^ L-101 50% pet. was applied) [10].

In view of the above, this multi-center study aimed to assess the prevalence of sensitization to lanolin alcohol 30% pet. and associated factors in North-Eastern Italy from 1997 through 2021. No data are available from Italy in this long timeframe, to the best of our knowledge.

## 2. Methods

This multi-centric cross-sectional study investigated sensitization to lanolin in 30,629 consecutive patients (67.5% females) patch tested during 1997–2021 (25 years) in various outpatients from North-Eastern Italy.

This study was approved by the local ethical committee of Friuli Venezia Giulia (CUER, protocol 092/2018) and written informed consent was obtained from all participating patients.

### 2.1. Study Population

The “*Triveneto patch database*” includes information on consecutive patch tests performed between 1997 and 2021 in various outpatient services of North-Eastern Italy. During the entire study period, the Triveneto patch test series, including 22 haptens (all in pet. when not otherwise specified), were employed (Supplementary Table S1).

All patients patch tested for present or past skin disease with signs and symptoms consistent with allergic CD were included in the present study. Patients with severe active skin diseases or those on immunosuppressant drugs were excluded [32].

### 2.2. Patients’ Evaluation

The clinical pattern of patients was assessed using the MOAHLFA Index (Males, Occupational dermatitis, Atopic dermatitis, Hand involvement, Leg involvement, Face involvement, Age > 40 years) [33]. Occupational CD was assessed by a consultant dermatologist or an occupational health consultant.

All patients were patch tested with Finn Chambers (Epitest, Tuusula, Finland) on Scanpor tape (Norgesplaster, Vennesla, Norway) and haptens produced by Chemotechnique Diagnostics (Vellinge, Sweden) and by FIRMA (Florence, Italy). European baseline series and the extended Triveneto series were used to patch test patients with suspected CD. All patches were applied on the upper part of patients’ backs and removed after 48 h. The area was examined upon removal of the patch (D2) and after 72/96 h (D3/D4), according to guidelines of the International Contact Dermatitis Research Group [25]:

- Reaction degrees +, ++ and +++ were considered positive.
- Uncertain responses (?+) were considered negative.

### 2.3. Statistical Analysis

Continuous variables were presented as mean ± standard deviation, median with interquartile range (IQR). Median were contrasted by Mann-Whitney test, whereas the chi-squared test was used to compare categorical variables.

Using administrative clerks as reference, univariable and multivariable logistic regression analysis was employed to investigate the risk of sensitization to lanolin, reporting odds ratio unadjusted (OR) and adjusted (aOR) for potential confounders, with 95% confidence interval (95%CI). Terms to be included in the final multivariable logistic regression model were selected by backward stepwise selection procedure.

The statistical analysis of the data was performed with STATA version 14.0 (Stata, College Station, TX, USA).

## 3. Results

**Figure 1:**
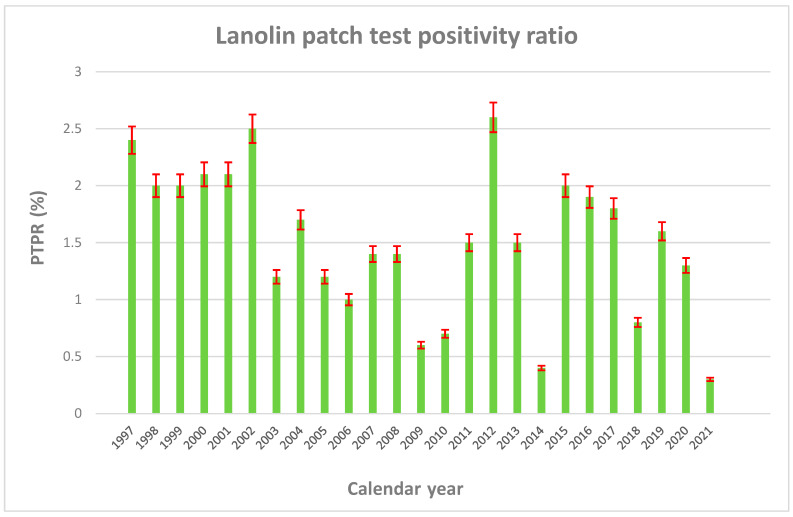
Frequency distribution of lanolin patch test positive ratio (PTPR) by calendar year (1997–2021). Error bars with whiskers are red marked.

Table 1 shows the distribution of patch tests performed by calendar year and research center. As can be noted, 30,629 patients with suspected allergic CD were patch tested against lanolin alcohol during 1997–2021 in the presents study, with some variability over time and by research center in terms of testing and PTPR. In particular, lanolin PTPR was rather higher in Pordenone until 2004. The temporal pattern of lanolin PTPR over time in the entire study population can be appreciated in Figure 1.

During the entire study period, the overall lanolin PTPR was 1.64% (=501/30,629), higher in the Province of Pordenone (2.17%) and Padua (1.89%), whereas it was lower in Trento-Bolzano-Rovigo (1.46%) and Trieste (1.05%). Lanolin PTPR was higher before 2005, especially in Pordenone, fluctuating thereafter (Table 1).

Table 2 displays the distribution of the study population by lanolin PTPR and explanatory terms as well as the results of logistic regression analysis. As can be noted, 501 (1.6%) patients were positive against lanolin out of 30,629 subjects patch tested. The median age of patients testing positive against lanolin was 42 (IQR: 30; 57) years and 60.7% (=304/501) of them were females.

The body area most frequently affected by CD were the hands (36.32%), followed by face (19.52%) and legs (8.09%), with a lanolin PTPR of 1.68%, 1.37% and 3.07%, respectively (Table 2).

Atopic dermatitis (AD) was identified in 2768 (10.1%) patients, with 1.2% (N = 33) of atopic patients testing positive against lanolin. Prevalence of occupational CD was 8.2%, with 1.8% (N = 46) patients with occupational CD patch testing positive to lanolin (Table 2).

At multiple logistic regression, the lanolin PTPR was significantly lower in the province of Trieste (aOR = 0.68; 95%CI: 0.52; 0.88) or Trento/Bolzano/Rovigo (aOR = 0.65; 95%CI: 0.45; 0.93) and higher in Pordenone (aOR = 1.38; 95%CI: 1.09; 1.75). Moreover, lanolin PTPR was significantly higher in males (aOR = 1.34; 95%CI: 1.09; 1.65), patients aged 38–48 years (aOR = 1.51; 95%CI: 1.08; 2.12), 48–60 years (aOR = 1.59; 95%CI: 1.12; 2.26), 61+ years (aOR = 1.94; 95%CI: 1.30; 2.91), those affected by leg CD (aOR = 1.67; 95%CI: 1.27; 2.19) or house painters (aOR = 7.56; 1.73; 33.15). Prevalence of lanolin sensitization significantly reduced over time (*p* < 0.001), especially after 2003 (Table 2).

Table 3 shows a further multiple logistic regression model fitted on the entire cohort and separately by sex of patients, adjusting for the same factors as the model displayed in Table 2, but including an interaction term between age and leg CD. As can be seen, the risk of lanolin sensitization was significantly higher among patients with leg CD aged 49–60 years (aOR = 2.34; 95%CI: 1.20; 4.57) or older than 60 (aOR = 4.21; 95%CI: 2.59; 6.85). Sub-group analysis by sex of patients confirmed the lower PTPR after 2002 for both sexes and the significantly higher sensitization rate in older patients with leg CD, with much stronger effect size in females 61+ years old (aOR = 5.33; 95%CI 2.87; 9.89) than males of the same age group (aOR = 2.92; 95%CI: 1.34; 6.39). Among patients without leg CD, males were more likely to test positive in the age band 49–60 years (aOR = 1.77; 95%CI: 1.01; 3.07), whereas females in the age band 38–48 years (aOR = 1.70; 95%CI: 1.11; 2.60). Moreover, house painting was significantly associated with lanolin sensitization exclusively in females, since no males in this occupational group tested positive.

## 4. Discussion

### 4.1. Key Findings

The present study examined sensitization to lanolin in a large group of consecutive patients (N = 30,629) of the “*Triveneto patch database*” (North-Eastern Italy) over the course of 25 years (1997–2021).

Five-hundred-and-one patients tested positive against lanolin, for a 1.47% PTPR in females versus 1.98% in males. The median age of patients testing positive to lanolin was 49 (IQR: 34; 63.5) years. The PTPR was higher in Pordenone (2.17%) and Padua (1.89%), yet lower in Trento-Bolzano-Rovigo (1.46%) and Trieste (1.05%).

The body area most frequently affected by CD were the hands (36.32%), but lanolin PTPR was higher in legs (3.07%) and patients with leg CD were more likely to test positive.

The rate of AD was 10.07% and 1.19% of atopic patients tested positive to lanolin.

Prevalence of occupational CD was 8.24%, with 1.83% of patients with occupational CD patch testing positive to lanolin.

Furthermore, lanolin PTPR significantly increased in patients with leg CD aged > 48—especially those older than 60—, in males or in house painters.

Sub-group analysis by sex confirmed the significantly higher sensitization rate of older patients with leg CD, with much stronger effect size in females 61+ years old than males in the same age band. Moreover, house painting was a risk factor for lanolin sensitization exclusively for females.

### 4.2. Interpretation of Findings

#### 4.2.1. Lanolin Patch Test Positivity Ratio

PTPR in the present study almost overlapped with that (1.65%) reported by a multi-center study of the European Surveillance System on Contact Allergies on 58,833 patients from 12 countries patch tested with lanolin alcohol 30% pet. during 2009–2012 [34]. Likewise, in an earlier single-center study from London (UK) on 24,449 patients patch tested with lanolin alcohol 30% pet. during 1982–1996, the mean PTPR was 1.7% (ranging between 0.9–2.3%) [35].

The variability of PTPR by research center, with higher rates until 2002 due to contributions of Padua and especially Pordenone, was hard to explain without additional information. A decreasing trend of sensitization against lanolin alcohol 30% petrolatum was also reported by an NACDG study on 26,479 patients patch-tested during 1994–2006, with a mean PTPR of 2.5%, decreasing from 3.7% in 1996–1998 to 1.8% through 2005–2006 [25]. Although PTPR was not negligible in the present study, it can be argued that the above variability, in line with variability in lanolin sensitization (0.45–6.29%) reported in the open literature [10,24,25,26,27], endorsed the ongoing controversy on the relevance of this hapten, which some authors have defined a myth or a “*paradox*” created by “*over-zealous patch testers*”, failing to consider the diagnostic limitations of the patch test procedure [22,36,37].

However, as already mentioned, much of this variability can be explained by the patch test formulation employed [10,24,25,26,27]. In particular, lanolin PTPR has been reportedly increasing in recent decades, as a likely result of multiple hapten derivatives being applied [10,26,29,30,31]. For instance, the above-mentioned NACDG study on 43,691 patients from North America conducted during 2011–2018 reported an overall 3.27% sensitization rate, increasing from 2.16% (=515/23,888) during 2001–2011 (when lanolin alcohol 30% pet. was applied) to 4.63% (=916/19,803) during 2011–2018 (when Amerchol^®^ L-101 50% pet. was used) [10].

There has been a long-standing debate on the patch test formulation to be used for lanolin, given the discrepancies between the three most used derivatives—lanolin alcohol 30% pet., Amerchol^®^ L-101 50% and Amerchol^®^ L-101 100% [13,26,28,30,31,35]. There is evidence that Amerchol^®^ L101 50% significantly increases the PTPR compared to lanolin alcohol 30% [10,26,28]. For instance, a recent large study on patients from the Information Network of Departments of Dermatology (IVDK) patch tested during 2006–2016 reported a crude PTPR of 3.48% (=2866/82,251) to Amerchol^®^ L-101 50% pet. (becoming 3.37% following age-sex standardization) against a crude 2.38% (=2762/115,885) PTPR to lanolin alcohols 30% pet. (becoming 2.35% following age–sex standardization). In the sub-group of patents patch tested against both haptens (N = 79,969), 2.05% were positive only to Amerchol^®^ L-101 50%, 1.19% only to lanolin alcohols 30% pet. and 1.43% to both haptens, for a total 4.67% PTPR to at least one allergen [29].

Considering the much lower concentration of wool wax alcohols in Amerchol^®^ L101 than lanolin alcohol 30% pet., false positive results secondary to the irritative effect on human skin by the former have been hypothesized [4,29].

Some authors claimed lanolin alcohol 30% pet. underestimates the true sensitization [30], while others stressed the importance to include Amerchol^®^ L-101 50% in the European standard series, similarly to North America, to maximize patch test sensitivity [28]. Since the latter approach may increase the rate of false positive results [10], repeated open application tests (ROAT) have been recommended on patients with unconvincing 1+ reactions to lanolin alcohols 30% pet. or Amerchol L-101 [4,13].

A debate is still open on the relevance of lanolin sensitization. Manufacturers of lanolin-containing products reported that lanolin used today is free from sensitizers and no longer a source of lanolin contact allergy [36,37]. A study contrasting 12 patients undergoing ROAT with lanolin-containing cream versus 14 controls, all consecutively patch tested to Amerchol L101 100% between 2009 and 2013 and exhibiting a ++ /+++ pattern, failed to induce any positive reaction [13]. The authors concluded that lanolin-containing emollients did not cause or worsen existing dermatitis in patients patch testing positive against Amerchol L101 100% [13].

#### 4.2.2. Male-to-Female Ratio

Despite 67.56% patients patch tested for suspected CD in the present study were females, lanolin PTPR was lower in females (1.47%) than males (1.98%), who were also more likely to test positive at multiple logistic regression analysis.

Regardless of the formulation employed, some studies reported a higher PTPR in females [35], while others in males [25,26,38] and others found no sex difference [28]. In the above study on 24,449 patients from London, 1.82% females tested positive to lanolin versus 1.63% males (*p* < 0.05) [35]. The above study on 43,691 patients patch tested with Amerchol L-101 50% found higher prevalence of sensitization in males [10]. Likewise, in a retrospective Dutch study on 9577 patients patch tested between 2004–2015, higher PTPR in males was observed against both Lanolin alcohol 30% pet. (36.2% = 21/58) and Amerchol™ L-101 50% pet. (39.2% = 31/79) [31]. It may be argued that Amerchol™ L-101 50% pet., which has been suspected of false positive results due to irritative action on human skin, may have a stronger sensitizing effect on female skin.

#### 4.2.3. Atopic Dermatitis

Similar to other studies [31,35], there was no evidence of an association between AD and lanolin sensitization in the present study. The association between AD and sensitization to lanolin is rather conflicting though. For instance, in a Dutch study on 594 patients patch tested during 2016–2017, AD was significantly associated with lanolin sensitization (OR = 1.75; 95%CI: 1.16; 2.63) [28]. Likewise, in another US study on 502 adults patch tested with expanded series between 2014–2017, atopic patients exhibited higher sensitization to ingredients used in emollients and topical medications, including Amerchol™ L-101 50% pet., whose PTPR was 4.6% in atopic patients vs. 1.3% in non-atopic (*p* = 0003) [39]. Finally, in the above NACDG study on 38,482 patients patch tested during 2011–2018 (using Lanolin alcohol 30% pet. until 2018 and Amerchol™ L-101 50% pet. afterwards), PTPR was significantly higher (*p* < 0.001) among 8336 patients with AD (3.8%) than in those 28,601 without it (2.3%) [10].

It can be argued that sensitization to lanolin in patients with AD could be attributable to personal care products containing the hapten.

#### 4.2.4. Leg Dermatitis and Age

**Figure 2:**
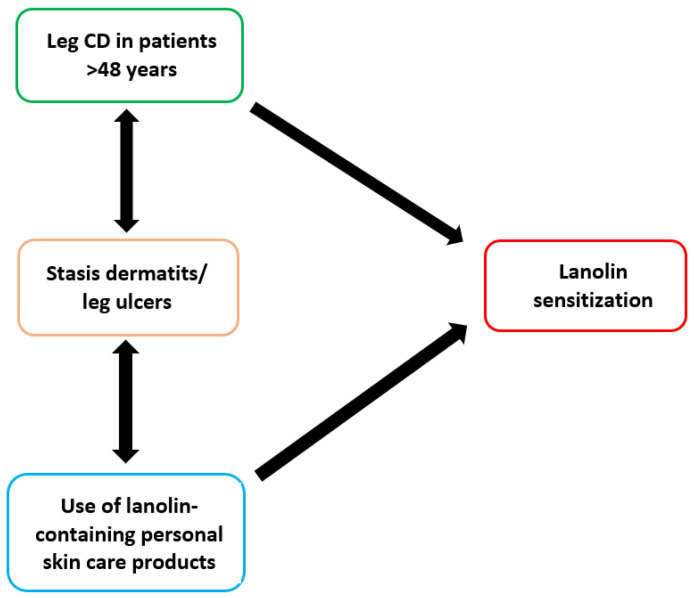
Conceptual framework displaying the potential confounding effect of lanolin skin care products on the association between leg contact dermatitis (CD) in patients older than 48 years with lanolin sensitization.

In the present study, patients with leg dermatitis exhibited significantly higher lanolin sensitization, especially those aged 61+ years, with a stronger effect size among females. Although no further specific information was available, it is known that stasis dermatitis increases the risk of sensitization to lanolin among older patients [40]. Leg dermatitis can in fact be secondary to stasis dermatitis or leg ulcers, conditions recommending topical application of emollients. Higher PTPR among females older than 60 with leg CD in the present study is in line with the hypothesis of exposure to lanolin-containing treatments against stasis dermatitis or leg ulcers (Figure 2).

In a multi-center study on 38,893 patients retrieved from 32 centers belonging to the IVDV network during 1996–1999—92.7% patch tested with lanolin alcohol 30% pet.—PTPR was 3.1% in those ≤ 60 years old, 8.3% in those aged 61–65 years and 12.5% in those >75 years old [40]. Stasis dermatitis strongly correlated with increased lanolin PTPR in older patients, although other risk factors may have been implicated [40].

Likewise, although the sensitization rate to 1+ allergen was significantly lower (40.7%) among 1444 patients with suspected CD older than 65 compared to corresponding patients aged 20–40 years (47.8%) in an Italian study, lanolin PTPR was significantly higher in the former group [41]. Adjusting for sex, age and history of atopic conditions, sensitization to lanolin alcohol 30% pet. was significantly higher in patients with leg ulcers older than 65 [41].

In another study on 235 patients with leg ulcers examined during 2001–2002—106 patch tested with the European standard series -, 75% had 1+ positive reaction, 57% had 2+ positive reactions and lanolin alcohol 30% pet. was the second most frequent allergen (21%) [42]. In an earlier study from the same group, conducted during 1997–2000 on 526 patients with dermatitis, 5.1% patch tested positive against lanolin and PTPR was significantly higher among patients with leg ulcers than in the general population [42].

In a multi-center study on 354 patients (226 females vs. 128 males) with leg ulcers treated with wound bandages, 59.6% had 1+ sensitization to one of the bandages and 19% had 1+ reaction to a component of the dressing [43]. Amerchol-L101 50% pet. was the second main allergen (5.4%) after benzalkonium chloride 0.1% pet. (7.1%) [43]. The number of positive test reactions per patient significantly increased with the duration of the condition causing the ulcer, but not with ulcer duration, cause of the ulcer or presence of surrounding eczematous lesions [43].

By contrast, duration of the leg ulcer, rather than its cause, significantly increased the number of positive patch tests per subject in a multi-center study including 423 patients (301 females vs. 122 males) [44]. The four main allergens at patch tests in the latter study were Myroxylon pereirae (40.7%), fragrance mix I (26.5%), Amerchol L-101 50% pet. (19.6%) and lanolin alcohol 30% pet. (17.7%) [44]. Likewise, Myroxylon pereirae (14.8%), fragrance mix I (11.4%), Amerchol L-101 50% pet. (9.7%) and lanolin alcohol 30% pet. (7.8%) were the four most frequent haptens among 5264 IVDK patients with leg ulcers associated with stasis dermatitis/chronic venous insufficiency patch tested during 2003–2014 [45]. Nonetheless, CD was more frequent among ulcer free controls (25.9%) than study subjects (16.9%) and sensitization to most haptens declined over time [45].

Taken together, all of the above evidence highly suggests a role of lanolin containing-personal care products used for stasis dermatitis or leg ulcers triggering sensitization to the hapten.

#### 4.2.5. Occupational Risk

**Figure 3:**
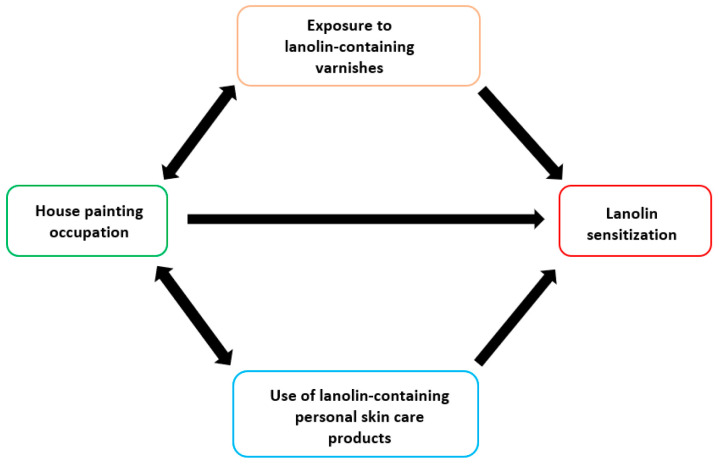
Conceptual framework displaying the potential confounding effect of use of lanolin-containing skin care products on the association between house painting occupation and lanolin sensitization.

Despite lanolin being mainly an extra-occupational hapten, an association between sensitization and house-painting occupation was found in the present study. Although the evidence, founded only on three patients—all females—could be easily confounded by use of lanolin-containing skin care products—including ointments to protect hands during house panting—occupational exposure to lanolin-containing varnishes shall not be ruled out (Figure 3), [46]. No further occupational group exhibited an increased risk of sensitization, despite lanolin also being used as a textile emollient, as a polish to protect tanned leather or to prevent corrosion of metal surfaces (e.g., to protect and preserve vehicles) or as lubricant for metal cutting oil, engineering parts or metal processing [46].

### 4.3. Strength and Weaknesses

The present is the largest study investigating lanolin sensitization in Italy over a long time period (25 years), employing a multi-center data collection, also assessing the impact of occupation and yielding adjusted prevalence estimates over time. However, the study limitations include the cross-sectional design, the variability of testing rate by research center and calendar year, and the lack of relevant definitions for lanolin sensitization. Furthermore, the cause of leg dermatitis and information on the eventual use of personal care products was not available.

## 5. Conclusions

Prevalence of lanolin sensitization was 1.64% in the present study, in line with the epidemiological pattern from other European settings. The body area most frequently affected by CD were the hands, although lanolin sensitization was higher in males and in patients with leg CD aged >37 years, probably due to use of creams and other personal skin care products containing lanolin for stasis dermatitis or leg ulcers, conditions typically increasing with age.

Lanolin sensitization was significantly higher in house painters, possibly due to exposure to lanolin-containing varnishes, although the latter association (limited to female patients) could be easily confounded by use of lanolin-containing skin care products.

Taken together, the above evidence endorses the ongoing debate on the relevance of lanolin skin reactions. Clinicians assessing patients with dermatitis should collect information on potential risk factors for lanolin sensitization, particularly use of skin care products containing the hapten. Occupational exposure to lanolin-containing varnishes should also be considered.

Future research should delve into exposure to lanolin among patients patch testing positive, especially the use of personal skin care products in older patients with stasis dermatitis and potential occupational exposure to the hapten, analyzing safety data sheets of varnishes or (if the latter information is not available) directly asking the producer.

## Figures and Tables

**Table 1:** Distribution of patch tests performed and lanolin patch test positivity ratio (PTPR) over time, by research centre. Frequency of tests performed (N. tests) and respective PTPR (row %).

Calendar Year	All Patients		Research Centre							
			Padua		Pordenone		Trieste		Trento/Bolzano/Rovigo	
	N. Tests	PTPR	N. Tests	PTPR	N. Tests	PTPR	N. Tests	PTPR	N. Tests	PTPR
1997	1242	2.42	395	2.03	445	4.04	398	1.01	4	0
1998	2566	2.03	1083	2.95	325	2.15	418	0.24	740	1.62
1999	2868	1.95	1189	2.94	278	2.52	453	0.44	948	1.27
2000	2445	2.13	1042	2.69	208	2.88	817	1.96	378	0.53
2001	2412	2.07	677	1.92	226	5.31	703	1.42	806	1.86
2002	1460	2.47	1	0	353	4.25	701	1.28	405	2.96
2003	1472	1.22	412	1.70	314	2.55	485	0.62	261	0
2004	842	1.66	287	1.74	94	6.38	370	0.81	91	0
2005	1135	1.15	434	1.61	383	1.31	318	0.31	0	NA
2006	1032	0.97	409	1.47	336	0.89	287	0.35	0	NA
2007	1115	1.35	439	1.14	368	2.45	308	0.32	0	NA
2008	1257	1.43	528	1.70	355	1.41	374	1.07	0	NA
2009	1112	0.63	328	0.61	373	0.27	411	0.97	0	NA
2010	1046	0.67	354	0.56	366	0.55	326	0.92	0	NA
2011	1107	1.45	234	2.14	377	1.59	496	1.01	0	NA
2012	664	2.56	0	0	286	3.85	378	1.59	0	NA
2013	886	1.47	240	1.25	274	2.19	372	1.08	0	NA
2014	1011	0.40	322	1.24	288	0	401	0	0	NA
2015	958	1.98	246	0.81	327	2.14	385	2.60	0	NA
2016	749	1.87	230	1.30	239	1.67	280	2.50	0	NA
2017	501	1.80	102	0.98	241	2.90	158	0.63	0	NA
2018	836	0.40	135	2.22	451	0.67	250	0.40	0	NA
2019	1008	1.59	381	0.26	211	4.27	416	1.44	0	NA
2020	540	1.30	94	0	265	1.89	181	1.10	0	NA
2021	365	0.27	0	NA	88	0	277	0.36	0	NA
**Total**	**501**	**1.64**	**3580**	**1.89**	**7471**	**2.17**	**9963**	**1.05**	**3633**	**1.46**

**Table 2:** Study population (N = 30,629) by patch test result against lanolin. Number (N), row percentage (%) and chi-squared *p*-value. Odds ratio unadjusted (OR) and adjusted (aOR) with 95% confidence interval (95%CI). CD = contact dermatitis; M = missing values. Obs. = complete case (analysis) observations.

Terms			Total Patients with CD N (col %)	Lanolin + N (row %)	*p*-Value	OR (95%CI)	aOR (95%CI) (25,907 obs.)
**Total patients examined for CD**			30,629 (100)	501 (1.64)			
**Centre**	**Padua**		9562 (31.22)	181 (1.89)	<0.001	*reference*	*reference*
	**Pordenone**		7471 (24.39)	162 (2.17)		1.15 (0.93;1.42)	1.38 (1.09; 1.75)
	**Trieste**		9963 (32.53)	105 (1.05)		0.55 (0.43; 0.70)	0.67 (0.52; 0.88)
	**Trento/Bolzano/Rovigo**		3633 (11.86)	53 (1.46)		0.77 (0.56; 1.04)	0.65 (0.45; 0.93)
**Sex**	**Females**		20,694 (67.56)	304 (1.47)	0.001	*reference*	*reference*
	**Males**		9935 (32.44)	197 (1.98)		1.36 (1.13; 1.63)	1.34 (1.09; 1.65)
**Age (years)** (M: 3)	**M ± SD**		43.80 ± 17.24	49.13 ± 18.50			
	**Median**		42 (30; 57)	49 (34; 63.5)	<0.001 *		
	**<28**		6315 (20.62)	73 (1.16)	<0.001	*reference*	*reference*
	**27–37**		6411 (20.93)	74 (1.15)		1.00 (0.72; 1.38)	1.01 (0.71; 1.45)
	**38–48**		6145 (20.06)	101 (1.64)		1.43 (1.06; 1.93)	1.51 (1.08; 2.12)
	**49–60**		5687 (18.57)	99 (1.74)		1.51 (1.12; 2.05)	1.59 (1.12; 2.26)
	**61+**		6068 (19.81)	153 (2.52)		2.21 (1.67; 2.93)	1.94 (1.30; 2.91)
**Atopic dermatitis** (M: 3143)	**No**		24,718 (89.93)	431 (1.74)	0.033	*reference*	
	**Yes**		2768 (10.07)	33 (1.19)		0.68 (0.48; 0.97)	
**Occupational** **dermatitis** (M: 31)	**No**		28,078 (91.76)	455 (1.62)	0.437	*reference*	
	**Yes**		2520 (8.24)	46 (1.83)		1.13 (0.83; 1.53)	
**Body area** **affected by CD**	**Hand** (M: 4432)	**No**	16,683 (63.68)	298 (1.79)	0.535	*reference*	
		**Yes**	9514 (36.32)	160 (1.68)		0.94 (0.77; 1.14)	
	**Leg** (M: 4430)	**No**	24,080 (91.1)	393 (1.63)	<0.001	*reference*	*reference*
		**Yes**	2119 (8.09)	65 (3.07)		1.91 (1.46; 2.49)	1.67 (1.27; 2.19)
	**Face** (M: 4430)	**No**	21,084 (80.48)	388 (1.84)	0.021	*reference*	
		**Yes**	5115 (19.52)	70 (1.37)		0.74 (0.57; 0.96)	
**Calendar year** **(1997–2021)**	**Linear term (1997–2021)**				<0.001	0.97 (0.96; 0.98)	
	**1997–2002**		9121 (29.78)	190 (2.08)	<0.001	*reference*	*reference*
	**2003–2007**		7321 (23.90)	131 (1.79)		0.58 (0.45; 0.76)	0.48 (0.35; 0.65)
	**2008–2012**		5562 (18.16)	57 (1.02)		0.58 (0.45; 0.77)	0.43 (0.31; 0.58)
	**2013–2017**		4626 (15.10)	69 (1.49)		0.67 (0.51; 0.89)	0.46 (0.34; 0.63)
	**2018–2021**		3999 (13.06)	54 (1.35)		0.53 (0.36; 0.76)	0.34 (0.22; 0.50)
**Occupation**	**Administrative clerks**		6692 (21.985)	87 (1.30)	<0.001	*reference*	*reference*
	**Health care workers**		3087 (10.08)	40 (1.30)		1.00 (0.68; 1.45)	0.89 (0.59; 1.33)
	**Teachers**		364 (1.19)	5 (1.37)		1.06 (0.43; 2.62)	1.31 (0.52; 3.30)
	**Cashiers**		26 (0.08)	0		-	-
	**Sellers**		353 (1.15)	2 (0.57)		0.43 (0.11; 1.76)	0.58 (0.14; 2.37)
	**Restaurant workers**		1297 (4.23)	23 (1.77)		1.37 (0.86; 2.18)	1.31 (0.80; 2.16)
	**Hairdressers**		388 (1.27)	1 (0.26)		0.20 (0.03; 1.41)	0.29 (0.04; 2.11)
	**Farmers**		257 (0.84)	6 (2.33)		1.81 (0.79; 4.19)	1.27 (0.50; 3.18)
	**Construction workers**		1178 (3.85)	20 (1.70)		1.31 (0.80; 2.14)	0.81 (0.47; 1.41)
	**House painters**		26 (0.08)	3 (11.54)		9.90 (2.92; 33.59)	7.56 (1.73; 33.15)
	**Painters, other**		76 (0.25)	1 (1.32)		1.01 (0.14; 7.36)	0.90 (0.12; 6.59)
	**Construction cleaners**		17 (0.06)	0		-	-
	**Mechanics**		1485 (4.85)	24 (1.62)		1.25 (0.79; 1.97)	1.03 (0.64; 1.66)
	**Workers of wood industry**		440 (1.44)	3 (0.68)		0.52 (0.16; 1.65)	0.38 (0.12; 1.22)
	**Artisan general**		454 (1.48)	9 (1.98)		1.54 (0.77; 3.07)	1.28 (0.61; 2.69)
	**Leather artisans**		114 (0.37)	0		-	-
	**Chemistry Industry workers**		229 (0.75)	4 (1.75)		1.35 (0.49; 3.71)	0.72 (0.22; 2.30)
	**Drivers**		280 (0.91)	5 (1.79)		1.38 (0.56; 3.43)	1.00 (0.40; 2.53)
	**Cleaners**		411 (1.34)	9 (2.19)		1.70 (0.85; 3.40)	1.89 (0.93; 3.83)
	**Housewives**		3564 (11.64)	69 (1.94)		1.50 (1.09; 2.06)	1.09 (0.75; 1.58)
	**Students**		830 (2.71)	11 (1.33)		1.02 (0.54; 1.92)	1.50 (0.75; 2.98)
	**Pensionants**		4394 (14.35)	119 (2.71)		2.11 (1.60; 2.79)	1.23 (0.83; 1.23)
	**Unemployed**		661 (2.16)	11 (1.66)		1.28 (0.68; 2.42)	1.26 (0.65; 2.46)
	**Other**		3847 (12.56)	49 (1.27)		0.98 (0.69; 1.39)	1.09 (0.74; 1.62)
	**Military**		159 (0.52)	0		--	-

* Mann-Whitney test.

**Table 3:** Distribution of the study population (N = 30,629) by patch test result against Lanolin (all patients; males; females). Number (N), row percentage (%) and chi-squared *p*-value. Odds ratio unadjusted (OR) and adjusted (aOR) with 95% confidence interval (95%CI). CD = contact dermatitis; M = missing values. Obs.= complete case (analysis) observations.

Terms.				All Patients		Males		Females	
				Lanolin + N (row %)	aOR (95%CI) (25,907 obs.)	Lanolin + N (row %)	aOR (95%CI) (8127 obs.)	Lanolin + N (row %)	aOR (95%CI) (17,513 obs.)
**Sex**		**Females**		304 (1.47)	*reference*				
		**Males**		197 (1.98)	1.34 (1.08; 1.65)				
**Leg CD** **(Interaction term)**	**No**	**Age** **(years)**	**<28**	73 (1.16)	*reference*	31 (1.58)	*reference*	42 (0.97)	*reference*
			**27–37**	74 (1.15)	1.02 (0.71; 1.47)	27 (1.27)	1.02 (0.57; 1.83)	47 (1.10)	1.00 (0.63; 1.59)
			**38–48**	101 (1.64)	1.50 (1.06; 2.12)	30 (1.55)	1.02 (0.56; 1.88)	71 (1.69)	1.70 (1.11; 2.60)
			**49–60**	99 (1.74)	1.53 (1.07; 2.19)	45 (2.41)	1.77 (1.01; 3.07)	54 (1.41)	1.32 (0.82; 2.12)
			**61+**	73 (1.16)	1.67 (1.10; 2.53)	64 (3.15)	1.71 (0.87; 3.36)	89 (2.21)	1.54 (0.90; 2.64)
	**Yes**	**Age** **(years)**	**<28**	3 (0.98)	0.78 (0.24; 2.50)	2 (1.71)	1.03 (0.24; 4.47)	1 (0.53)	0.50 (0.07; 3.68)
			**27–37**	2 (0.66)	0.57 (0.14; 2.36)	0	1.05 (0.20; 5.51)	2 (1.03)	0.99 (0.24; 4.19)
			**38–48**	7 (1.79)	1.63 (0.73; 3.62)	3 (2.00)	1.48 (0.43; 5.07)	4 (1.66)	1.66 (0.58; 4.77)
			**49–60**	11 (2.82)	2.34 (1.20: 4.57)	6 (3.17)	2.14 (0.84; 5.48)	5 (2.49)	2.35 (0.89; 6.20)
			**61+**	42 (5.75)	4.21 (2.59; 6.85)	16 (4.94)	2.92 (1.34; 6.39)	26 (6.40)	5.33 (2.87; 9.89)
**Occupation**		**Administrative**		87 (1.30)	*reference*	38 (1.84)	*reference*	49 (1.06)	*reference*
		**House painters**		3 (11.54)	7.69 (1.75; 33.76)	0	NA	3 (14.29)	10.92 (2.39; 49.90)
**Calendar year**		**1997–2002**		190 (2.08)	*reference*	80 (2.71)	*reference*	110 (1.78)	*reference*
		**2003–2007**		131 (1.79)	0.49 (0.36; 0.66)	46 (1.90)	0.49 (0.30; 0.81)	85 (1.73)	0.48 (0.32; 0.72)
		**2008–2012**		57 (1.02)	0.43 (0.32; 0.59)	23 (1.30)	0.48 (0.30; 0.78)	34 (0.90)	0.41 (0.27; 0.61)
		**2013–2017**		69 (1.49)	0.47 (0.34; 0.64)	31 (215)	0.47 (0.29; 0.79)	38 (1.19)	0.48 (0.32; 0.71)
		**2018–2021**		54 (1.35)	0.34 (0.22; 0.51)	17 (1.26)	0.30 (0.16; 0.59)	37 (1.40)	0.37 (0.22; 0.61)

All three regression models are adjusted for research center, sex, age, leg CD, occupation and calendar year.

## Data Availability

The data generated and analyzed during the current study are not publicly available since they were purposively collected by the authors for the present study, but they are available from the corresponding author upon reasonable request.

## Supplementary Materials

The following supporting information can be downloaded at: https://www.mdpi.com/article/10.3390/life14080916/s1, Supplementary Table S1: “Triveneto patch test series (22 haptens) tested in the overall study period (all in pet. when not otherwise specified)”; Supplementary Figure S1: “Lanolin patch test positivity ratio (PTPR) by research center, 1997–2021”.
