# Supplementary material for: Sensitization to Lanolin in North-Eastern Italy, 1997–2021: Prevalence, Risk Factors and the Impact of Occupation
Source: Life (Basel). 2024 Jul 23;14(8):916. doi: 10.3390/life14080916 (PMC11355248; doi:10.3390/life14080916)
Supplement: Supplementary file 1 [file life-14-00916-s001.zip › life-3110493-supplementary.pdf]

Supplementary Table S1. Triveneto patch test series (22 haptens) tested in the overall study period (all in pet when not otherwise specified)

**1**	4-ter-Butylphenol- formaldehyde resin 1%
**2**	Carba mix 3%
**3**	Cobalt chloride hexahydrate 1%
**4**	Colophonium 20%
**5**	Disperse blu 35 1%
**6**	Disperse yellow 3 1%
**7**	Epoxy resin 1%
**8**	Formaldehyde 1% aq
**9**	Fragrance mix-I 8%
**10**	Methyl-chloro-isothiazolinon/methyl-isothiazolinon (Kathon) 0.02 aq.
**11**	Lanolin alcohol 30%,
**12**	Mercaptobenzothiazole 2%
**13**	Mercaptobenzothiazole mix
**14**	Neomycin sulfate 20%
**15**	Nickel sulphate 5%
**16**	N-Isopropy-N-phenyl-4-phenylendiamine 0.1%
**17**	Parabens mix
**18**	Peru balsam 25%
**19**	Potassium bichromate 0.5%
**20**	p-Phenylenediamine 1%
**21**	Quaternium-15 1%
**22**	Thiuram mix 1%

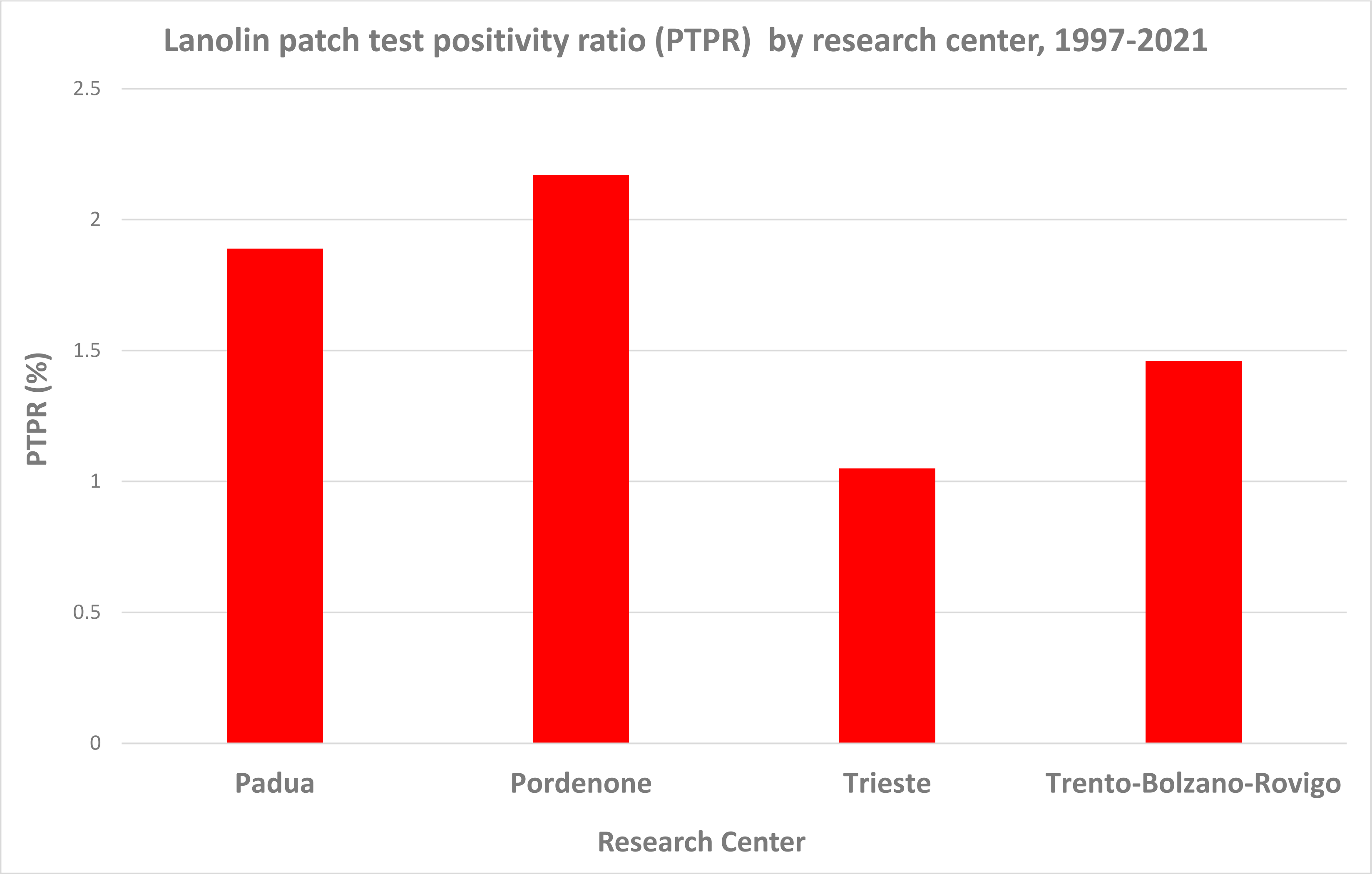

Supplementary Figure S1. Lanolin patch test positivity ratio (PTPR) by research center, 1997-2021.
